# Mitochondria-Targeting Small Molecules Effectively Prevent Cardiotoxicity Induced by Doxorubicin

**DOI:** 10.3390/molecules23061486

**Published:** 2018-06-19

**Authors:** Wei Shi, Hongkuan Deng, Jianyong Zhang, Ying Zhang, Xiufang Zhang, Guozhen Cui

**Affiliations:** 1 Department of Bioengineering, Zhuhai Campus of Zunyi Medical University, Zhuhai 519041, China; diana1354726@outlook.com (W.S.); Zhangying@hotmail.com (Y.Z.); 2 School of Life Sciences, Shandong University of Technology, Zibo 255000, China; hongkuandeng@gmail.com (H.D.); zhangxiufang5@163.com (X.Z.); 3 Pharmacy School, Zunyi Medical University, Zunyi 563003, China; zhangjianyong2006@126.com

**Keywords:** doxorubicin, cardiotoxicity, mitochondria, small molecules

## Abstract

Doxorubicin (Dox) is a chemotherapeutic agent widely used for the treatment of numerous cancers. However, the clinical use of Dox is limited by its unwanted cardiotoxicity. Mitochondrial dysfunction has been associated with Dox-induced cardiotoxicity. To mitigate Dox-related cardiotoxicity, considerable successful examples of a variety of small molecules that target mitochondria to modulate Dox-induced cardiotoxicity have appeared in recent years. Here, we review the related literatures and discuss the evidence showing that mitochondria-targeting small molecules are promising cardioprotective agents against Dox-induced cardiac events.

## 1. Introduction

Cancer is a serious public health problem and the second leading cause of death in the world [1]. Cancer chemotherapy has made significant progress in the treatment of both solid and hematologic malignancies [2]. Anthracycline chemotherapy plays an important role in the modern era of cancer treatment [3]. Doxorubicin (Dox) is an anthracycline antibiotic drug that has been widely used to treat various types of tumors, such as leukemia and solid tumors [4]. Unfortunately, its clinical use has been greatly limited by dose-dependent and cumulative cardiotoxicity [5,6]. Sometimes, even at low doses of Dox exposure (200–250 mg/m^2^), cardiotoxic events still may occur in 10% of patients [7]. Recent studies demonstrated that the onset of the cardiotoxicity may be delayed more than 10 years after cessation of Dox chemotherapy [8].

Currently, the precise mechanisms underlying Dox-induced cardiotoxicity have not been fully understood but are likely to be multiple. In particular, overgeneration of reactive oxygen species (ROS) and inhibition of topoisomerase-II have been implicated as key mechanisms of Dox-induced cardiotoxicity [9,10]. Additionally, accumulated studies have suggested that Dox-induced mitochondrial dysfunction is a major cause of Dox-induced cardiotoxicity [11,12]. Mitochondria are among the key intracellular sites for the production of active free radical intermediates [9,13]. Dox has a high affinity for cardiolipin, which is involved in the reactions and processes of mitochondrial biogenesis. Dox binds to cardiolipin then enters the mitochondria and inhibits the respiratory chain [14]. Other proposed mitochondrial cardiotoxicity mechanisms include impaired expression of sundry important cardiac proteins [15]; mitochondrial membrane potential rapid depolarization; induction of mitochondrial DNA damage [16], destruction of mitochondrial bioenergetics [17]; metabolism of Dox into cardiotoxic and more hydrophilic substances; disruption of cellular and mitochondrial Ca^2+^ homeostasis [18]; and interference with different kinds of pro-survival kinases [19]. Therefore, it seems clear that mitochondrial maintenance plays an important role in preventing Dox-induced cardiotoxicity. In recent years, the use of small molecules targeting mitochondria to prevent Dox-induced cardiotoxicity has been extensively investigated and has made considerable progress. Although several reviews on small molecules to attenuate Dox-induced cardiotoxicity have been published in recent years, they mainly focus on the reduction of Dox-induced cardiotoxicity by phenolic compounds [20,21], plant-derived small molecules [22], and some natural products [23]. One issue was still unclear: the common mechanism underlying this action exerted by most of the small molecules. In this review, for the first time, we summarize the findings about the possible mode of actions of 42 small molecules that target mitochondria to reduce Dox-induced cardiotoxicity.

## 2. Small Molecules that Attenuate Dox-Induced Cardiotoxicity

In this section, we systematically describe the myocardial protection for small molecules against Dox-induced cardiotoxicity. They are divided into three categories: natural products, semisynthetic small molecules, and synthetic compounds. A brief description of their effects on anti-tumor activity when co-treated with Dox and their roles in pathways or targets are listed in Table 1.

### 2.1. Natural Products

#### 2.1.1. Plant-Derived Small Molecules

Arjunolic acid: Arjunolic acid (AA, 2,3,23-trihydroxyolean-12-en-28-oic acid) is a naturally occurring triterpenoid saponin with various biological functions, including antioxidant [111], hepatoprotective [112], and antifungal properties [113]. The effect of AA on Dox-induced cardiac abnormalities in a rat model and an oxidative stress model was investigated, and the results showed that AA treatment attenuated Dox-induced apoptosis in heart tissue, and could block Dox-induced disrupted MMP (mitogen-activated protein kinase), mitochondria-mediated caspase-dependent apoptosis, and enhanced cell injury in cardiomyocytes [24].

Baicalein: Baicalein (5,6,7-trihydroxy-2-phenyl-4*H*-1-benzopyran-4-one) is a natural flavonoid derived from *Scutellariae radix*. Due to its antioxidant potential, in vitro models demonstrated that it could protect cardiomyocytes against ischemia–reperfusion, lysophosphatidylcholine and oxidative stress-induced cell injury [25,26,28]. Another study by Chang et al. indicated that baicalein could attenuate Dox-induced cardiotoxicity not only by attenuation of mitochondrial oxidant injury, but also by suppression of c-Jun NH2-terminal kinase (JNK) activation. Interestingly, treatment of baicalein did not compromise its anticancer efficacy [27].

Berberine: Berberine (Ber) is an isoquinoline alkaloid with various biological effects, including anti-tumor and cardiovascular protection [114]. Ber inhibits Dox-induced cardiomyocyte apoptosis by reducing mitochondrial dysfunction and increasing bcl-2 expression [30,34]. Ber significantly reduces mitochondrial Ca^2+^ overload, increases myocardial energy metabolism, and inhibits Dox-induced acute changes in myocardial calcium homeostasis [29]. In addition, Ber can also inhibit the metabolism of Dox into doxorubicinol (a secondary alcohol metabolite) and exert its cardioprotective effect [31]. Doxorubicinol is stable in the heart and accumulates there to produce a long-lasting poison [33]. Studies have shown that combined Ber with Dox treatment can significantly inhibit cancer cell proliferation [32].

Curcumin: Curcumin, a yellow pigment, is a major component of turmeric. It is reported that turmeric may control Dox-mediated cardiotoxicity and decrease oncogenesis [115]. Several studies have shown that curcumin could protect against Dox-induced cardiotoxicity. The key mechanisms postulated for the cardioprotective activity of curcumin include induction of apoptosis, JNK activation, abrogation of inflammation, and excessive opening of mitochondrial permeability transition pores [35,36,37,38,39,40]. Therefore, pre-treatment with curcumin could be considered in cancer chemotherapeutic applications.

Cryptotanshinone: Cryptotanshinone (CRY) is one of the major constituents of *Salvia miltiorrhiza*, which has been widely used to treat various diseases such as myocardial infarction, angina pectoris, and ischemic stroke [116]. In adult male Wistar rats, the administering of CRY could prevent doxorubicin-induced cardiotoxicity by increasing mitochondrial biogenesis and enhancing the activities of the mitochondrial respiratory chain complex [41]. 

Chrysin: Chrysin, a flavone, is present in honey, mushrooms, and bee propolis [117]. Traditionally, chrysin is used to enhance testosterone concentration. Healthy human volunteers consumed daily doses of up to 2–3 g without side effects [118]. A recent study in rats demonstrated that chrysin could effectively protect against Dox-induced cardiomyopathy. The mechanism appeared to involve suppressing oxidative stress, the mitochondrial apoptotic pathway, MAPK (mitogen-activated protein kinase), and NF-κB (nuclear factor kappa-B) pathways [42].

Cyclovirobuxine D (CVB-D) is a triterpene alkaloid extracted from a traditional Chinese herb, cloves. A study in rats demonstrated that CVB-D has a certain therapeutic effect on heart failure induced by myocardial infarction [119]. CVB-D could ameliorate Dox-induced cardiomyopathy in mice by inhibiting Dox-induced mitochondrial cytochrome c release and mitochondrial biosynthesis damage [43].

Cannabidiol: Cannabidiol (CBD) is a non-psychotropic cannabinoid that has strong antioxidant and anti-inflammatory effects [120]. Studies in experimental models showed it could protect against Dox-induced heart damage [121]. In rat model experiments, CBD may reduce the infarct size of the ischemia–reperfusion model by reducing the inflammatory response [44]. CBD prevented Dox-induced cardiomyopathy/heart failure in mice by reducing oxidative stress and mitochondrial dysfunction and promoting mitochondrial biogenesis [13].

Esculetin: Esculetin is a natural phenolic in *Cortex fraxini*. It is known to have multiple functions including anticancer and neuroprotective activity [122,123]. Recently, an in vitro study found that esculetin could prevent against Dox-induced cardiotoxicity; the mechanism of the effect is involved in the enhanced Bmi-1 expression, suppression of ROS accumulation and mitochondrial damage, and subsequently reducing Dox-induced cell apoptosis [45].

Honokiol: Honokiol (HKL), a polyphenol derived from the magnolia tree, protected against Dox-induced cardiac hypertrophy in mice through enhancing mitochondrial function by activating Sirt3 without compromising the anti-tumor activity of Dox [46]. A line of evidence showed that HKL attenuated Dox-induced cardiotoxicity by increasing mitochondrial protein acetylation as well as activating PPARγ in a mouse heart [47].

Hydroxytyrosol: Hydroxytyrosol (HT) is a polyphenol composition of leaf oil with a wide variety of beneficial effects on human health, especially its protection against cardiovascular diseases, cancer, and metabolic disorders [124,125]. The protective effects of HT against Dox-induced cardiotoxicity have been investigated in rats. The results demonstrated that HT could effectively ameliorate Dox-induced heart damage by improving the mitochondrial electron transport chain and oxidative damage [48].

Isorhamnetin: Isorhamnetin is a naturally occurring flavonoid and can reduce Dox-induced oxidative stress and inhibit the activation of mitochondrial apoptotic pathway and MAPK pathway. It is worth noting that isorhamnetin attenuated cardiac damage without compromising the anticancer efficacy in vivo. Isorhamnetin also improves the anticancer activity of Dox in MCF-7, HepG2, and Hep2 cells [49]. 

Kaempferol: Kaempferol is a polyphenolic compound widely found in food from plants. It has anti-inflammatory [126], antioxidative [127], and anticancer effects [128]. Studies have shown that Kaempferol may participate in the ERK (extracellular signal-regulated kinase)-dependent MAPK pathway to reduce Dox-induced cardiac toxicity by inhibiting p53-mediated mitochondrial-dependent intrinsic apoptotic signaling [51]. A recent study showed that Kaempferol combined with Dox or cisplatin has a strong synergistic therapeutic effect on HCT-15 and MDA-MB-231 cells [50].

Luteolin-7-*O*-Glucoside: Luteolin-7-*O*-Glucoside (LUTG) is a flavonoid isolated from the whole plant of *Aspergillus flavus* and has a protective effect against Dox-induced cardiotoxicity [129]. A study by Yao et al. demonstrated that LUTG reversed mitochondrial depolarization and decreased apoptosis in H9C2 cells [52]. It was reported that luteolin has a biphasic effect on the survival rate of the human breast cancer cell line MCF-7. That is, luteolin displayed a cell proliferative effect at low concentrations (10 μM), but a cytotoxic effect at high concentrations (above 30 μM) [53].

Myricitrin: Myricitrin, a natural flavonoglycoside, has displayed multiple beneficial biological activities including anti-allergic effects and anxiolytic action [130,131]. In vitro and in vivo studies have demonstrated that myricitrin could significantly attenuate Dox-induced cardiotoxicity. The underlying mechanism is involved in the antioxidant activity and its inhibition of mitochondria-dependent apoptotic signaling [54].

Naringin: Naringin (4′,5,7-trihydroxyflavanone-7-rhamnoglucoside) is present in grapefruit juice and has metal chelating and antioxidant properties [132]. Naringin was effective at reducing the oxidative stress induced by Dox in the liver of mice [133]. In vivo and in vitro studies have demonstrated that Naringin provided mitochondrial protection by inhibiting MAPK expression and ROS generation, without compromising its antineoplastic activity [55,56].

Oxymatrine: Oxymatrine (OMT) is an active ingredient of the traditional Chinese herb *Sophora flavescens*. OMT has a protective effect on aldosterone-mediated injuries by inhibiting apoptosis-inducing factor signaling pathways and calpain [134]. In vivo and in vitro studies have shown that OMT reduced oxidative stress, leading to decreased cardiac apoptosis and subsequent changes to myocardial architecture [57]. OMT has a pro-apoptotic effect in human breast cancer MCF-7 cells [58]; further study is needed to determine whether the combination of OMT and Dox interferes with its anti-tumor activity.

Ophiopogonin D: Ophiopogonin D (OP-D), an active component of *Ophiopogon japonicas*, has been reported to confer protecting endothelial cells from oxidative stress-induced cell injury [135]. Recently, in vitro and mice model studies demonstrated that OP-D exerted a cardioprotective effect against Dox-induced autophagic cell death by relieving ROS-induced mitochondrial impairment [59].

Plantainoside D: Plantainoside D (PD) is an active component isolated from the plant *Picrorhiza scrophulariiflora* with potential anti-hypertensive function [134]. A recent study by Kim et al. demonstrated that PD could also protect Dox-induced apoptosis in H9c2 cardiomyoblast cells. The mechanism of the protective effect can be explained by the inhibition of ROS production and NF-κB activation [60].

Quercetin: Quercetin is a polyphenolic flavonoid present in many fruits, vegetables, and grains with a wide variety of health benefits, including its pharmacological ability to lower blood pressure [136] and protect the brain, heart, and liver against various factors related to oxidative stress [61,62,63]. An in vitro and in vivo study showed that quercetin could effectively inhibit Dox-induced cardiotoxicity and mitochondrial dysfunction by upregulation of Bmi-1 expression [64].

Resveratrol: Resveratrol (RV) is present in a variety of food skins including grapes, mulberries, and blueberries. The experimental model in H9c2 cells demonstrated that it could prevent Dox-induced cardiotoxicity via inhibition of cell injury, mitochondrial stabilization, specifically the activation of the Sirt1 pathway [65,67]. Furthermore, RV enhanced cardiac function and prevented oxidant stress responses in rats [66].

Rosmarinic acid: Rosmarinic acid (RA) is a water-soluble natural phenolic compound that is isolated from the rosemary plant and has a high content in the Labiatae and the Boraginaceae families. RA could ameliorate cardio-nephrotoxicity induced by Dox in rats through their anti-inflammatory, antioxidant, and anti-apoptotic activities [68]. It was also found to exhibit inhibitory effects on Dox-induced apoptosis in H9c2 cardiomyocytes by inhibiting the activations of ROS, JNK and extracellular signal-regulated kinases [69].

Sesamin: Sesamin (Ses) is one of the main active ingredients in sesame seeds and has multiple pharmacological functions, including hepatoprotection, cholesterol-lowering, and cardiovascular protective properties [137,138,139]. The pre-clinical evidence demonstrated that Ses could also protect cardiac tissue and H9c2 cells against Dox-induced cardiac injury. The major underlying mechanism of this effect is contributed to Sirt1 activation [70].

Sulforaphane: Sulforaphane, a natural compound present in cruciferous vegetables, is a potent Nrf2 inducer. The study in mice demonstrated that sulforaphane treatment significantly enhanced the activity of the mitochondrial respiratory complex and exhibited protective effects against Dox-induced cardiotoxicity [71]. Additionally, a recent study in rats and neonatal rat cardiomyocytes demonstrated that sulforaphane could not only protect the heart against Dox-induced toxicity via protection of mitochondrial function and integrity, but also synergistically exhibited an anti-tumor effect with Dox [72].

Salvianolic acid A: *Salvia miltiorrhiza* is widely used in the treatment of cardiovascular diseases in China. Salvianolic acid A (SAI) is the main bioactive component of *Salvia miltiorrhiza*. In addition, the antioxidant effect of SAI suggests that it protects the heart through blocking oxidative stress [74]. Several studies have indicated that SAI can inhibit Dox-induced myocardial mitochondrial lipid peroxidation, while it has no antagonizing effect on the anti-tumor activity of Dox [73].

Tetrandrine: Tetrandrine is an alkaloid derivative isolated from the roots of the *Stephania tetrandra* plant. The experimental study in a rat model found that tetrandrine has protective potential in Dox-induced cardiotoxicity. The underlying mechanisms of the effect were involved in the protective effects against Dox-induced impairment of mitochondrial oxidative phosphorylation and oxidative phosphorylation [75].

2,3,5,4′-tetrahydroxystilbene-2-*O*-β-d-glucoside (THSG), one of the active ingredients of the traditional anti-aging drug *Polygonum multiflorum*, has strong antioxidant [140] and anti-inflammatory effects [141]. Experimental studies have demonstrated that THSG protects Dox-induced cardiotoxicity by reducing ROS production and intracellular Ca^2+^, inhibiting apoptotic pathways, reducing mitochondrial membrane potential rapid depolarization and dysfunction [77]. Previous reports have demonstrated that THSG inhibits DNA synthesis and lung metastasis in tumor cells [76].

Visnagin: Visnagin naturally occurs in a kind of flowering plant, *Ammi visnaga*, which has been used as a herbal medicine to treat kidney stones [142]. Visnagin could protect Dox-induced cardiac dysfunction in zebrafish and mice without reducing the chemotherapeutic efficacy of Dox in vitro. The further mechanism study demonstrated that the inhibition of mitochondrial malate dehydrogenase (MDH2) is responsible for the cardioprotection of visnagin [78]. Interestingly, an editorial suggested that further exploration is warranted to develop protective agents against Dox cardiotoxicity in the clinic [79].

#### 2.1.2. Others

a-Linolenic acid: a-Linolenic acid (ALA) is an essential dietary fatty acid that cannot be synthesized by the body itself [143]. ALA could inhibit oxidative stress and reduce the damage of acute myocardial ischemia–reperfusion injury in the heart [144]. An in vivo study showed that ALA could protect the heart against Dox-induced cardiotoxicity by regulating mitochondrial apoptosis pathways and inhibiting oxidative stress [81]. Compared with free Dox, Dox conjugate ALA showed a good anti-tumor effect and low toxicity in terms of longevity, inhibition of tumor growth, and body weight changes [80].

All-trans retinoic acid: All-trans retinoic acid (ATRA) is one of the major bioactive products of vitamin A metabolism. ATRA is a differentiation agent of cancer stem cells [145]. The combination of ATRA, low molecular weight heparin, and Dox exerts a stronger anti-tumor effect and significantly reduces side effects [146]. A study found that ATRA could protect against Dox-induced cardiotoxicity in H9c2 cells and primary cardiomyocytes by activating the ERK2 signaling pathway and restoring mitochondrial function [82]. A recent study in rats demonstrated that ATRA could also ameliorate Dox-induced cardiotoxicity. The mechanism of the protective effect can be explained by antioxidative and anti-inflammatory properties [83].

BAY60-2770: BAY 60-2770, a soluble guanylyl cyclase (SGC) activator, has been shown to ameliorate the impairment of urethral relaxation in obese mice [147]. An in vivo and in vitro study demonstrated that BAY 60-2270 has a chemosensitization effect on cancer and the potential to decrease Dox anti-tumor resistance [84]. BAY 60-2270 reduces Dox-induced mitochondrial membrane potential loss by upregulating mitochondrial ferritin expression [85].

Ghrelin: Ghrelin is a multifaceted gut hormone mostly produced by the stomach. In addition to its function in mediating energy homeostasis, ghrelin also has a powerful cardioprotective effect [148], and was shown to regulate the secretion of insulin [149]. Ghrelin and Des-acyl ghrelin have protective effects against chemotherapy-induced cardiotoxicity [88]. The principal finding of the studies was that ghrelin maintains mitochondrial shapes, reduces autophagic vacuoles [87], and can prevent mitochondrial bioenergetic dysfunction and reduce mitochondrial pathways of apoptosis [86].

Melatonin: Melatonin, a natural endocrine hormone, is non-toxic and is both a sedative and a cardioprotective agent [150]. Melatonin prevents the mitochondrial damage induced by Dox in mouse fibroblasts, while melatonin co-treatment with Dox increased the PPAR-gamma and AMPK levels. Melatonin could protect cardiomyocytes against Dox’s cytotoxicity by increasing cell proliferation and inhibiting apoptosis [90]. In addition, we have demonstrated that melatonin inhibits mitochondrial ROS production and preserves mitochondrial membrane potential during Dox-induced cardiotoxicity [89].

### 2.2. Semisynthetic Small Molecules

D006: D006 is a new danshensu (DSS) derivative, derived from coupling the structures of DSS, TMP (tetramethylpyrizine), and ACS (4-(3-thioxo-3*H*-1, 2-dithiol-4-yl)-benzoic acid). Recently, an in vivo and in vitro study showed that D006 has cardioprotective effects against Dox-induced cardiotoxicity. The mechanism may be mediated by the promotion of HO-1 protein expression and the preservation of mitochondrial biogenesis. D006 also enhanced Dox-induced breast cancer cell apoptosis through p53 activation [91].

Mitochondrial division inhibitor: Mitochondrial division inhibitor (Mdivi-1) is a derivative of quinazolinone. It was identified as the most efficacious inhibitor during chemical screening of mitochondrial division inhibitors; it selectively inhibits the Dynamin-related protein 1 (Drp1 is required for mitochondrial division) [151]. Mdivi-1 inhibited mitochondrial fission and played an important role in ameliorating heart failure [93]. A study by Gharanei et al. demonstrated that Mdivi-1 reversed Dox-induced depolarization of cardiac myocytes and reduced myocardial infarct size without affecting its anticancer properties [92].

Sodium tanshinone IIA sulphonate (STS): STS, a water-soluble derivative of tanshinone IIA, has anti-inflammatory [152] and protective effects on myocardial ischemia [153]. In vitro and in vivo experimental data demonstrated that STS acts as a protective agent against Dox-induced cardiotoxicity [154]. STS has been shown to reduce cardiotoxicity by attenuating Dox-induced mitochondrial lipid peroxidation and swelling [94,95].

### 2.3. Synthetic Compounds

Bafilomycin A1, rapamycin: Bafilomycin A1 and rapamycin are traditional autophagy inducers. Recent studies have shown that macroautophagy is involved in Dox-induced cardiotoxicity [155]. The study indicated that Bafilomycin A1 and rapamycin attenuate the cardiotoxic effects of Dox inH9c2 cells as well as in a breast tumor-bearing mouse model. The result indicated that Bafilomycin A1 or rapamycin could effectively reduce the total production of mitochondrial ROS, increase mitochondrial function, and resist Dox-induced toxicity [96].

Diazoxide: Diazoxide, a molecular formula of C_8_H_7_ClN_2_O_2_S, has powerful protective properties against cardiac ischemia [156]. An in vivo study demonstrated that diazoxide protects against Dox-induced cardiotoxicity through open mitochondrial K_ATP_ channels [97,98]. Recently, the cardioprotective effects of diazoxide against Dox-induced cardiotoxicity were further confirmed by enhancing the expression of mitochondrial connexin 43 [99].

Dexrazoxane (Dxz): Dxz, is the only drug approved by the FDA to prevent Dox-induced cardiotoxicity. Dxz significantly reduces Dox-induced cardiotoxicity in pediatric solid tumor patients [157]. Dxz limits heart damage by chelating free iron, and reduces mitochondrial iron levels [11]. The results from an animal study indicated that Dxz can ameliorate Dox-induced cardiac injury by protecting myocardial mitochondria from genetic and functional impairment [100,101].

Metformin (Met): Met is an effective and safe oral drug for the treatment of type II diabetes. A study indicated that Met supplementation could attenuate the conduction abnormalities and mechanical dysfunction caused by Dox in rats [158]. Met plays an important role in cardiomyocytes by regulating the expression of transcription factor NF-κB, thereby protecting cardiomyocytes against Dox-induced oxidative stress and apoptosis [102]. Recently, Elashmawy et al. showed that Met combined with Dox has a marked anti-tumorigenic effect [103]. A recent study in human breast carcinoma (MCF7/ADR) cells suggested that Met can be used for the treatment of chemotherapy-resistant tumors, and can restore the sensitivity of Dox [104].

Nicorandil: Nicorandil, a mitochondrial K_ATP_ channel opener and nitric oxide donor, has been administered to successfully counteract the toxic effects of Dox [159]. Nicorandil was effective in ameliorating Dox-induced heart failure in rats [105]. The underlying mechanism is the activation of the mitochondrial ATP-sensitive K^+^ channel, causing mitochondrial depolarization, inhibition of mitochondrial NADPH oxidase, and mitochondrial ultrastructural changes [106,107].

Sildenafil: Sildenafil is a PDE-5 inhibitor that can reduce Dox-induced apoptosis, depletion of pro-survival proteins such as bcl-2, and dissipation of mitochondrial membrane potential (ΔΨm) [108]. In addition, sildenafil increased eNOS/NOS in the heart and opened the mitochondrial K_ATP_ channels, thereby improving the cardiac contractile function that was impaired by Dox [109]. Interestingly, the combination of Dox and sildenafil can enhance the anti-tumor activity of Dox [110]. The molecular mechanisms underlying the different responses of the combined treatment to application of cardiomyocytes and tumor cells remain unclear. Undoubtedly, it would be an interesting issue that is worth investigating in the future.

## 3. Conclusions and Future Directions

In recent years, the mitochondria-related mechanisms of chemotherapeutic drug-induced cardiotoxicity have been extensively studied, and Dox is one of them [160]. Accumulated evidence shows that small molecules in pre-clinical and clinical studies have proven to be effective in preventing this toxicity. However, most of the experiments have not been translated into clinical trials in humans, which are required to provide explicit information about the efficacy of these small molecules.

Aside from the protective efficacy of small molecules against Dox-induced cardiotoxicity, it is significant to ensure that the anticancer effects of Dox are not minimized by a combined use of small molecules. This question has been addressed in a number of studies, and we can conclude from Table 1 that baicalein, visnagin, Mdivi-1, bafilomycin A1, Dxz, and nicorandil did not influence the anti-tumor activity of Dox, Interestingly, berberine, isorhamnetin, kaempferol, sulforaphane, tetrandrine, THSG, ATRA, melatonin, D006, Met, and sildenafil not only attenuated cardiotoxicity, but also potentiated anti-tumor activity in various models. 

**Figure 1:**
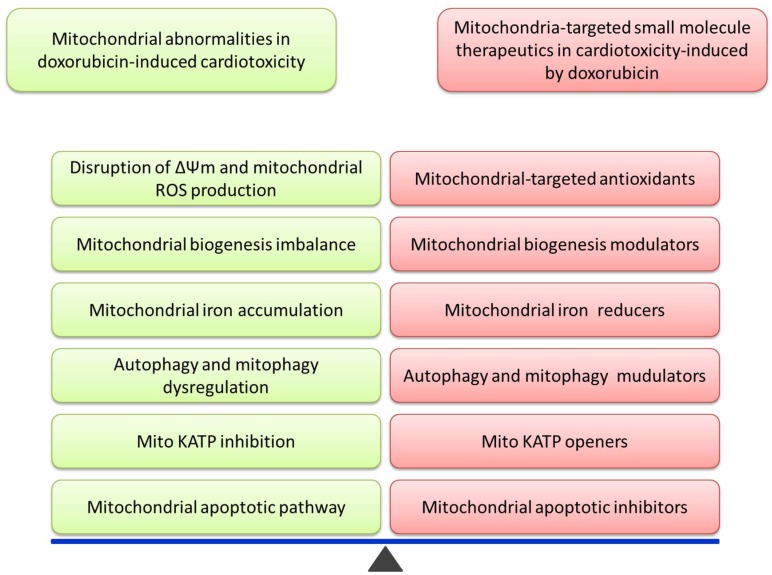
This image shows that establishing mitochondrial stability by small molecules is a critical step of potential therapeutic mechanisms in Dox-induced cardiotoxicity.

Here, we summarized the potential small molecules that can be used to limit the cardiotoxic effect of Dox. We have found that the potential utilities of small molecules targeting mitochondria deserve further studies in order to develop them into potential therapeutic agents for protecting thousands of cancer patients against Dox-induced cardiotoxicity while undergoing chemotherapy. Small molecules play a key role in maintaining mitochondrial stability from different aspects. (Figure 1). Understanding the effects and the underlying mechanisms of the combined utilization will help guide future efforts in developing agent modulators against Dox-associated cardiotoxicity. In the future, mitochondrial directed therapies involving small molecules will open up novel avenues for the management of mitochondrial function, providing protection from Dox-related cardiotoxicity.

## Figures and Tables

**Table 1:** Small molecules that target mitochondria effectively prevent the cardiotoxicity induced by Dox.

Name of Molecules	Model	Key Mechanisms of the Action Against Dox	Anti-Cancer Effect	Refs.
AA	NRC, rats	↓Disruption of ΔΨm	-	[24]
		↓Mitochondrial apoptotic pathway		
Baicalein	Chick cardiomyocytes	↓Disruption of ΔΨm	↔	[25,26,27,28]
		↓ROS		
		↓JNK activation		
Berberine	NRC, MCF-7 cells, rats	↓Mitochondrial dysfunction	↑	[29,30,31,32,33,34]
		↓Disruption of ΔΨm		
		↓Mitochondrial apoptotic pathway		
		↓Mitochondrial Ca^2+^		
		↓Dox metabolize		
Curcumin	Rats, Mice H9c2	↑Mitochondrial K_ATP_ channel	-	[35,36,37,38,39,40]
		↓Mitochondrial phosphate carrier		
		↓Mitochondrial superoxide radicals		
CRY	Rats	↑Mitochondrial biogenesis	-	[41]
		↑Activities of mitochondrial respiratory chain complex		
Chrysin	Rats	↓Mitochondrial apoptotic pathway	-	[42]
		↓MAPK and NF-κB activation		
		↑VEGF/AKT pathway		
CVB-D	Mice	↑Mitochondrial biogenesis	-	[43]
CBD	Mice, rats	↑Mitochondrial function	-	[13,44]
		↑Mitochondrial biogenesis		
		↓Pro-inflammatory response		
Esculetin	H9c2	↑Mitochondrial function	-	[45]
		↑Bmi-1 expression		
		↓ROS		
HKL	Mice	↑Cardiac mitochondrial respiration	↔	[46,47]
		↑Sirt3		
		↑PPARγ		
HT	Rats	↑Mitochondrial dysfunction	-	[48]
		↑Mitochondrial electron transport chain		
Isorhamnetin	H9c2, rats, MCF-7, HepG2 and Hep2	↓Mitochondria-dependent apoptotic Pathway	↑	[49]
		↓MAPK pathway		
		↓ROS		
Kaempferol	H9c2, rats	↓Mitochondrial dysfunction	↑	[50,51]
		↓Disruption of ΔΨm		
		↓Mitochondrial apoptotic pathway		
LUTG	H9c2	↓Disruption of ΔΨm	↕	[52,53]
Myricitrin	H9c2, rats	↓Disruption of ΔΨm	-	[54]
		↓Mitochondrial apoptotic pathway		
		↓ROS		
Naringin	H9c2, rats	↓Disruption of ΔΨm	-	[55,56]
		↓P38 MAPK		
		↓ROS		
OMT	H9c2, rats	↓Mitochondrial apoptotic pathway	-	[57,58]
		↓ROS		
OP-D	H9c2, mice	↓Disruption of ΔΨm	-	[59]
		↓Autophagy and ROS		
PD	H9c2	↓Disruption of ΔΨm	-	[60]
		↓ROS		
		↓NF-κB activation		
Quercetin	H9c2, mice	↓Mitochondrial dysfunction	-	[61,62,63,64]
		↓Disruption of ΔΨm		
		↓ROS		
		↑Bmi-1 expression		
RV	NRC	↓Disruption of ΔΨm	-	[65,66,67]
		↑Sirt1 pathway		
		↓ROS		
RA	H9c2	↓Disruption of ΔΨm	-	[68,69]
		↓ROS		
Ses	H9c2, rats	↓Disruption of ΔΨm	-	[70]
		↑Sirt1 and Mn-SOD pathway		
Sulforaphane	H9c2, NRC, rats	↑Nrf2	↑	[71,72]
		↓Disruption of ΔΨm		
		↓Mitochondrial apoptotic pathway		
SAI	Rats	↓Membrane sclerosis	↔	[73,74]
	L1210 cells			
Tetrandrine	Rats	↑Mitochondrial function	↑	[75]
		↓Mitochondrial oxidative phosphorylation		
THSG	Mice, NRC	↓Disruption of ΔΨm	↑	[76,77]
		↓Mitochondrial apoptotic pathway		
		↓ROS		
Visnagin	Zebrafish, Mice, NRC, HL1, MCF7, DU145, LNCaP, MDA-MB-231	↓Mitochondrial malate dehydrogenase 2 activity	↔	[78,79]
ALA	Rats	↓Mitochondrial apoptotic pathway	-	[80,81]
		↑Nrf2		
ATRA	H9c2	↑Mitochondrial function	↑	[82,83]
		↓Mitochondrial biogenesis damage		
BAY60-2770	H9c2, rats	↓ROS	-	[84,85]
		↓Disruption of ΔΨm		
		↑Mitochondrial ferritin		
Ghrelin	NRC, H9c2, mice	↓Disruption of ΔΨm	-	[86,87,88]
		↑mitochondrial bioenergetics		
		↓Mitochondrial apoptotic pathway		
Melatonin	H9c2, rats	↑Mitochondrial biogenesis	↑	[89,90]
	NIH3T3 cells	↑PPARγ		
		↓ROS		
D006	H9c2, zebrafish	↓mitochondrial biogenesis	↑	[91]
	MCF-7			
Mdivi-1	Rats, NRC, HL60	↓Mitochondrial fission	↔	[92,93]
STS	Mice, Rats	↓Mitochondrial lipid peroxidation and swelling	-	[94,95]
Bafilomycin A1, rapamycin	H9c2, mice	↑Autophagy	↔	[96]
		↓ROS		
		↑Mitochondrial function		
Diazoxide	Rats, mice	↑Mitochondrial K_ATP_ channel	-	[97,98,99]
		↑Mitochondrial connexin		
Dxz	NRC, Rats	↓Mitochondrial iron accumulation	↔	[11,100,101]
	Mice	↓Mitochondrial DNA		
Met	Mice, rats	↑Mitochondrial function	↑	[102,103,104]
	HL-1	↓Mitochondrial apoptotic pathway		
	MCF7/ADR			
Nicorandil	Rats, HL-1	↑Mitochondrial function	↔	[105,106,107]
		↓Mitochondrial apoptotic pathway		
		↑Mitochondrial creatine kinase activity and oxidative phosphorylation capacity		
		↑Mitochondrial K_ATP_ channel		
Sildenafil	Mice, mouse cardiomyocytes	↑Mitochondrial K_ATP_ channel	↑	[108,109,110]
		↓Disruption of ΔΨm		

↑, increase or open; ↓, decrease or inhibit; ↔, no difference; -, no description; ↕, biphasic effect; ΔΨm, mitochondrial membrane potential; NRC, Neonatal rat cardiomyocytes.

## References

[1] Siegel R.L., Miller K.D., Jemal A. (2018). Cancer statistics, 2018. Cancer J. Clin..

[2] Volkova M., Russell R. (2011). Anthracycline cardiotoxicity: Prevalence, pathogenesis and treatment. Curr. Cardiol. Rev..

[3] McGowan J.V., Chung R., Maulik A., Piotrowska I., Walker J.M., Yellon D.M. (2017). Anthracycline Chemotherapy and Cardiotoxicity. Cardiovasc. Drugs Ther..

[4] Liu H., Wang H., Xiang D., Guo W. (2017). Pharmaceutical Measures to Prevent Doxorubicin-Induced Cardiotoxicity. Mini Rev. Med. Chem..

[5] Sterba M., Popelova O., Vavrova A., Jirkovsky E., Kovarikova P., Gersl V., Simunek T. (2013). Oxidative stress, redox signaling, and metal chelation in anthracycline cardiotoxicity and pharmacological cardioprotection. Antioxid. Redox Signal..

[6] Li D.L., Wang Z.V., Ding G., Tan W., Luo X., Criollo A., Xie M., Jiang N., May H., Kyrychenko V. (2016). Doxorubicin Blocks Cardiomyocyte Autophagic Flux by Inhibiting Lysosome Acidification. Circulation.

[7] Bernstein D. (2018). Anthracycline Cardiotoxicity: Worrisome Enough to Have You Quaking?. Circ. Res..

[8] Armstrong G.T., Joshi V.M., Ness K.K., Marwick T.H., Zhang N., Srivastava D., Griffin B.P., Grimm R.A., Thomas J., Phelan D. (2015). Comprehensive Echocardiographic Detection of Treatment-Related Cardiac Dysfunction in Adult Survivors of Childhood Cancer: Results From the St. Jude Lifetime Cohort Study. J. Am. Coll. Cardiol..

[9] Zhang S., Liu X., Bawa-Khalfe T., Lu L.S., Lyu Y.L., Liu L.F., Yeh E.T. (2012). Identification of the molecular basis of doxorubicin-induced cardiotoxicity. Nat. Med..

[10] Kim S.Y., Kim S.J., Kim B.J., Rah S.Y., Chung S.M., Im M.J., Kim U.H. (2006). Doxorubicin-induced reactive oxygen species generation and intracellular Ca2+ increase are reciprocally modulated in rat cardiomyocytes. Exp. Mol. Med..

[11] Ichikawa Y., Ghanefar M., Bayeva M., Wu R., Khechaduri A., Naga Prasad S.V., Mutharasan R.K., Naik T.J., Ardehali H. (2014). Cardiotoxicity of doxorubicin is mediated through mitochondrial iron accumulation. J. Clin. Investig..

[12] Jain D. (2000). Cardiotoxicity of doxorubicin and other anthracycline derivatives. J. Nucl. Cardiol. Off. Publ. Am. Soc. Nucl. Cardiol..

[13] Hao E., Mukhopadhyay P., Cao Z., Erdelyi K., Holovac E., Liaudet L., Lee W.S., Hasko G., Mechoulam R., Pacher P. (2015). Cannabidiol Protects against Doxorubicin-Induced Cardiomyopathy by Modulating Mitochondrial Function and Biogenesis. Mol. Med..

[14] Wallace K.B. (2003). Doxorubicin-induced cardiac mitochondrionopathy. Pharmacol. Toxicol..

[15] Boucek R.J., Miracle A., Anderson M., Engelman R., Atkinson J., Dodd D.A. (1999). Persistent effects of doxorubicin on cardiac gene expression. J. Mol. Cell. Cardiol..

[16] Lebrecht D., Kokkori A., Ketelsen U.P., Setzer B., Walker U.A. (2005). Tissue-specific mtDNA lesions and radical-associated mitochondrial dysfunction in human hearts exposed to doxorubicin. J. Pathol..

[17] Tokarska-Schlattner M., Zaugg M., Zuppinger C., Wallimann T., Schlattner U. (2006). New insights into doxorubicin-induced cardiotoxicity: The critical role of cellular energetics. J. Mol. Cell. Cardiol..

[18] Solem L.E., Henry T.R., Wallace K.B. (1994). Disruption of mitochondrial calcium homeostasis following chronic doxorubicin administration. Toxicol. Appl. Pharmacol..

[19] Peng X., Chen B., Lim C.C., Sawyer D.B. (2005). The cardiotoxicology of anthracycline chemotherapeutics: Translating molecular mechanism into preventative medicine. Mol. Interv..

[20] Razavi-Azarkhiavi K., Iranshahy M., Sahebkar A., Shirani K., Karimi G. (2016). The Protective Role of Phenolic Compounds Against Doxorubicin-induced Cardiotoxicity: A Comprehensive Review. Nutr. Cancer.

[21] Mattera R., Benvenuto M., Giganti M.G., Tresoldi I., Pluchinotta F.R., Bergante S., Tettamanti G., Masuelli L., Manzari V., Modesti A. (2017). Effects of Polyphenols on Oxidative Stress-Mediated Injury in Cardiomyocytes. Nutrients.

[22] Ojha S., Al Taee H., Goyal S., Mahajan U.B., Patil C.R., Arya D.S., Rajesh M. (2016). Cardioprotective Potentials of Plant-Derived Small Molecules against Doxorubicin Associated Cardiotoxicity. Oxid. Med. Cell. Longev..

[23] Yu J., Wang C., Kong Q., Wu X., Lu J.J., Chen X. (2018). Recent progress in doxorubicin-induced cardiotoxicity and protective potential of natural products. Phytomed. Int. J. Phytother. Phytopharmacol..

[24] Ghosh J., Das J., Manna P., Sil P.C. (2011). The protective role of arjunolic acid against doxorubicin induced intracellular ROS dependent JNK-p38 and p53-mediated cardiac apoptosis. Biomaterials.

[25] Cui G., Luk S.C., Li R.A., Chan K.K., Lei S.W., Wang L., Shen H., Leung G.P., Lee S.M. (2015). Cytoprotection of baicalein against oxidative stress-induced cardiomyocytes injury through the Nrf2/Keap1 pathway. J. Cardiovasc. Pharmacol..

[26] Chen H.M., Hsu J.H., Liou S.F., Chen T.J., Chen L.Y., Chiu C.C., Yeh J.L. (2014). Baicalein, an active component of Scutellaria baicalensis Georgi, prevents lysophosphatidylcholine-induced cardiac injury by reducing reactive oxygen species production, calcium overload and apoptosis via MAPK pathways. BMC Complement. Altern. Med..

[27] Chang W.T., Li J., Haung H.H., Liu H., Han M., Ramachandran S., Li C.Q., Sharp W.W., Hamann K.J., Yuan C.S. (2011). Baicalein protects against doxorubicin-induced cardiotoxicity by attenuation of mitochondrial oxidant injury and JNK activation. J. Cell. Biochem..

[28] Shao Z.H., Vanden Hoek T.L., Qin Y., Becker L.B., Schumacker P.T., Li C.Q., Dey L., Barth E., Halpern H., Rosen G.M. (2002). Baicalein attenuates oxidant stress in cardiomyocytes. Am. J. Physiol. Heart Circ. Physiol..

[29] Xiong C., Wu Y.Z., Zhang Y., Wu Z.X., Chen X.Y., Jiang P., Guo H.C., Xie K.R., Wang K.X., Su S.W. (2018). Protective effect of berberine on acute cardiomyopathy associated with doxorubicin treatment. Oncol. Lett..

[30] Wang Y., Liu J., Ma A., Chen Y. (2015). Cardioprotective effect of berberine against myocardial ischemia/reperfusion injury via attenuating mitochondrial dysfunction and apoptosis. Int. J. Clin. Exp. Med..

[31] Hao G., Yu Y., Gu B., Xing Y., Xue M. (2015). Protective effects of berberine against doxorubicin-induced cardiotoxicity in rats by inhibiting metabolism of doxorubicin. Xenobiotica Fate Foreign Compd. Biol. Syst..

[32] Mittal A., Tabasum S., Singh R.P. (2014). Berberine in combination with doxorubicin suppresses growth of murine melanoma B16F10 cells in culture and xenograft. Phytomed. Int. J. Phytother. Phytopharmacol..

[33] Salvatorelli E., Menna P., Gonzalez Paz O., Surapaneni S., Aukerman S.L., Chello M., Covino E., Sung V., Minotti G. (2012). Pharmacokinetic characterization of amrubicin cardiac safety in an ex vivo human myocardial strip model. II. Amrubicin shows metabolic advantages over doxorubicin and epirubicin. J. Pharmacol. Exp. Ther..

[34] Lv X., Yu X., Wang Y., Wang F., Li H., Wang Y., Lu D., Qi R., Wang H. (2012). Berberine inhibits doxorubicin-triggered cardiomyocyte apoptosis via attenuating mitochondrial dysfunction and increasing Bcl-2 expression. PLoS ONE.

[35] Mohajeri M., Sahebkar A. (2018). Protective effects of curcumin against doxorubicin-induced toxicity and resistance: A review. Crit. Rev. Oncol./Hematol..

[36] Benzer F., Kandemir F.M., Ozkaraca M., Kucukler S., Caglayan C. (2018). Curcumin ameliorates doxorubicin-induced cardiotoxicity by abrogation of inflammation, apoptosis, oxidative DNA damage, and protein oxidation in rats. J. Biochem. Mol. Toxicol..

[37] Jain A., Rani V. (2018). Mode of treatment governs curcumin response on doxorubicin-induced toxicity in cardiomyoblasts. Mol. Cell. Biochem..

[38] Lu J., Chu E., Tony H., Li X., Sudeep K.C., Zhang M., Wang Y., Qi X.Q. (2016). Curcumin Downregulates Phosphate Carrier and Protects against Doxorubicin Induced Cardiomyocyte Apoptosis. Biomed. Res. Int..

[39] Katamura M., Iwai-Kanai E., Nakaoka M., Okawa Y., Ariyoshi M., Mita Y., Nakamura A., Ikeda K., Ogata T., Ueyama T. (2014). Curcumin Attenuates Doxorubicin-Induced Cardiotoxicity by Inducing Autophagy via the Regulation of JNK Phosphorylation. J. Clin. Exp. Cardiol..

[40] Imbaby S., Ewais M., Essawy S., Farag N. (2014). Cardioprotective effects of curcumin and nebivolol against doxorubicin-induced cardiac toxicity in rats. Hum. Exp. Toxicol..

[41] Zhang Y., Chen L., Li F., Wang H., Yao Y., Shu J., Ying M.Z. (2016). Cryptotanshinone protects against adriamycin-induced mitochondrial dysfunction in cardiomyocytes. Pharm. Biol..

[42] Mantawy E.M., Esmat A., El-Bakly W.M., Salah ElDin R.A., El-Demerdash E. (2017). Mechanistic clues to the protective effect of chrysin against doxorubicin-induced cardiomyopathy: Plausible roles of p53, MAPK and AKT pathways. Sci. Rep..

[43] Guo Q., Guo J., Yang R., Peng H., Zhao J., Li L., Peng S. (2015). Cyclovirobuxine D Attenuates Doxorubicin-Induced Cardiomyopathy by Suppression of Oxidative Damage and Mitochondrial Biogenesis Impairment. Oxid. Med. Cell. Longev..

[44] Durst R., Danenberg H., Gallily R., Mechoulam R., Meir K., Grad E., Beeri R., Pugatsch T., Tarsish E., Lotan C. (2007). Cannabidiol, a nonpsychoactive Cannabis constituent, protects against myocardial ischemic reperfusion injury. Am. J. Physiol. Heart Circ. Physiol..

[45] Xu F., Li X., Liu L., Xiao X., Zhang L., Zhang S., Lin P., Wang X., Wang Y., Li Q. (2017). Attenuation of doxorubicin-induced cardiotoxicity by esculetin through modulation of Bmi-1 expression. Exp. Ther. Med..

[46] Pillai V.B., Kanwal A., Fang Y.H., Sharp W.W., Samant S., Arbiser J., Gupta M.P. (2017). Honokiol, an activator of Sirtuin-3 (SIRT3) preserves mitochondria and protects the heart from doxorubicin-induced cardiomyopathy in mice. Oncotarget.

[47] Huang L., Zhang K., Guo Y., Huang F., Yang K., Chen L., Huang K., Zhang F., Long Q., Yang Q. (2017). Honokiol protects against doxorubicin cardiotoxicity via improving mitochondrial function in mouse hearts. Sci. Rep..

[48] Granados-Principal S., El-Azem N., Pamplona R., Ramirez-Tortosa C., Pulido-Moran M., Vera-Ramirez L., Quiles J.L., Sanchez-Rovira P., Naudi A., Portero-Otin M. (2014). Hydroxytyrosol ameliorates oxidative stress and mitochondrial dysfunction in doxorubicin-induced cardiotoxicity in rats with breast cancer. Biochem. Pharmacol..

[49] Sun J., Sun G., Meng X., Wang H., Luo Y., Qin M., Ma B., Wang M., Cai D., Guo P. (2013). Isorhamnetin protects against doxorubicin-induced cardiotoxicity in vivo and in vitro. PLoS ONE.

[50] Kalyani C., Narasu M.L., Devi Y.P. (2017). Synergistic growth inhibitory effect of flavonol–kaempferol and conventional chemotherapeutic drugs on cancer cells. Int. J. Pharm. Pharm. Sci..

[51] Xiao J., Sun G.B., Sun B., Wu Y., He L., Wang X., Chen R.C., Cao L., Ren X.Y., Sun X.B. (2012). Kaempferol protects against doxorubicin-induced cardiotoxicity in vivo and in vitro. Toxicology.

[52] Yao H., Shang Z., Wang P., Li S., Zhang Q., Tian H., Ren D., Han X. (2016). Protection of Luteolin-7-O-Glucoside Against Doxorubicin-Induced Injury Through PTEN/Akt and ERK Pathway in H9c2 Cells. Cardiovasc. Toxicol..

[53] Sato Y., Sasaki N., Saito M., Endo N., Kugawa F., Ueno A. (2015). Luteolin attenuates doxorubicin-induced cytotoxicity to MCF-7 human breast cancer cells. Biol. Pharm. Bull..

[54] Sun J., Sun G., Cui X., Meng X., Qin M., Sun X. (2016). Myricitrin Protects against Doxorubicin-Induced Cardiotoxicity by Counteracting Oxidative Stress and Inhibiting Mitochondrial Apoptosis via ERK/P53 Pathway. Evid. Based Complement. Altern. Med..

[55] Jian C.Y., Ouyang H.B., Xiang X.H., Chen J.L., Li Y.X., Zhou X., Wang J.Y., Yang Y., Zhong E.Y., Huang W.H. (2017). Naringin protects myocardial cells from doxorubicininduced apoptosis partially by inhibiting the p38MAPK pathway. Mol. Med. Rep..

[56] Kwatra M., Kumar V., Jangra A., Mishra M., Ahmed S., Ghosh P., Vohora D., Khanam R. (2016). Ameliorative effect of naringin against doxorubicin-induced acute cardiac toxicity in rats. Pharm. Biol..

[57] Zhang Y.Y., Yi M., Huang Y.P. (2017). Oxymatrine Ameliorates Doxorubicin-Induced Cardiotoxicity in Rats. Cell. Physiol. Biochem. Int. J. Exp. Cell. Physiol. Biochem. Pharmacol..

[58] Lin B., Li D., Zhang L. (2016). Oxymatrine mediates Bax and Bcl-2 expression in human breast cancer MCF-7 cells. Die Pharm..

[59] Zhang Y.Y., Meng C., Zhang X.M., Yuan C.H., Wen M.D., Chen Z., Dong D.C., Gao Y.H., Liu C., Zhang Z. (2015). Ophiopogonin D attenuates doxorubicin-induced autophagic cell death by relieving mitochondrial damage in vitro and in vivo. J. Pharmacol. Exp. Ther..

[60] Kim D.S., Woo E.R., Chae S.W., Ha K.C., Lee G.H., Hong S.T., Kwon D.Y., Kim M.S., Jung Y.K., Kim H.M. (2007). Plantainoside D protects adriamycin-induced apoptosis in H9c2 cardiac muscle cells via the inhibition of ROS generation and NF-kappaB activation. Life Sci..

[61] Lei X., Chao H., Zhang Z., Lv J., Li S., Wei H., Xue R., Li F., Li Z. (2015). Neuroprotective effects of quercetin in a mouse model of brain ischemic/reperfusion injury via anti-apoptotic mechanisms based on the Akt pathway. Mol. Med. Rep..

[62] Ji L.L., Sheng Y.C., Zheng Z.Y., Shi L., Wang Z.T. (2015). The involvement of p62-Keap1-Nrf2 antioxidative signaling pathway and JNK in the protection of natural flavonoid quercetin against hepatotoxicity. Free Radic. Biol. Med..

[63] Liu H., Guo X., Chu Y., Lu S. (2014). Heart protective effects and mechanism of quercetin preconditioning on anti-myocardial ischemia reperfusion (IR) injuries in rats. Gene.

[64] Dong Q., Chen L., Lu Q., Sharma S., Li L., Morimoto S., Wang G. (2014). Quercetin attenuates doxorubicin cardiotoxicity by modulating Bmi-1 expression. Br. J. Pharmacol..

[65] Li Y.G., Zhu W., Tao J.P., Xin P., Liu M.Y., Li J.B., Wei M. (2013). Resveratrol protects cardiomyocytes from oxidative stress through SIRT1 and mitochondrial biogenesis signaling pathways. Biochem. Biophys. Res. Commun..

[66] Tatlidede E., Sehirli O., Velioglu-Ogunc A., Cetinel S., Yegen B.C., Yarat A., Suleymanoglu S., Sener G. (2009). Resveratrol treatment protects against doxorubicin-induced cardiotoxicity by alleviating oxidative damage. Free Radic. Res..

[67] Danz E.D., Skramsted J., Henry N., Bennett J.A., Keller R.S. (2009). Resveratrol prevents doxorubicin cardiotoxicity through mitochondrial stabilization and the Sirt1 pathway. Free Radic. Biol. Med..

[68] Maher O.W., Raslan Y.A., Ahmed A.A.E., Raafat E.M., Georgy G.S. (2016). The Ameliorative Effect of Ellagic Acid and Rosemarinic Acid against Cardio-nephrotoxicity Induced by Doxorubicin in Rats. Int. J. Sci. Res. Publ..

[69] Kim D.S., Kim H.R., Woo E.R., Hong S.T., Chae H.J., Chae S.W. (2005). Inhibitory effects of rosmarinic acid on adriamycin-induced apoptosis in H9c2 cardiac muscle cells by inhibiting reactive oxygen species and the activations of c-Jun N-terminal kinase and extracellular signal-regulated kinase. Biochem. Pharmacol..

[70] Su S., Li Q., Liu Y., Xiong C., Li J., Zhang R., Niu Y., Zhao L., Wang Y., Guo H. (2014). Sesamin ameliorates doxorubicin-induced cardiotoxicity: Involvement of Sirt1 and Mn-SOD pathway. Toxicol. Lett..

[71] Singh P., Sharma R., McElhanon K., Allen C.D., Megyesi J.K., Benes H., Singh S.P. (2015). Sulforaphane protects the heart from doxorubicin-induced toxicity. Free Radic. Biol. Med..

[72] Bose C., Awasthi S., Sharma R., Benes H., Hauer-Jensen M., Boerma M., Singh S.P. (2018). Sulforaphane potentiates anticancer effects of doxorubicin and attenuates its cardiotoxicity in a breast cancer model. PLoS ONE.

[73] Lin T.J., Liu G.T., Liu Y., Xu G.Z. (1992). Protection by salvianolic acid A against adriamycin toxicity on rat heart mitochondria. Free Radic. Biol. Med..

[74] Jiang B., Zhang L., Li M., Wu W., Yang M., Wang J., Guo D.A. (2008). Salvianolic acids prevent acute doxorubicin cardiotoxicity in mice through suppression of oxidative stress. Food Chem. Toxicol. Int. J. Publ. Br. Ind. Biol. Res. Assoc..

[75] Xu M., Sheng L., Zhu X., Zeng S., Chi D., Zhang G.J. (2010). Protective effect of tetrandrine on doxorubicin-induced cardiotoxicity in rats. Tumori.

[76] Kimura Y., Okuda H. (2000). Effects of naturally occurring stilbene glucosides from medicinal plants and wine, on tumour growth and lung metastasis in Lewis lung carcinoma-bearing mice. J. Pharm. Pharmacol..

[77] Zhang S.H., Wang W.Q., Wang J.L. (2009). Protective effect of tetrahydroxystilbene glucoside on cardiotoxicity induced by doxorubicin in vitro and in vivo. Acta Pharmacol. Sin..

[78] Liu Y., Asnani A., Zou L., Bentley V.L., Yu M., Wang Y., Dellaire G., Sarkar K.S., Dai M., Chen H.H. (2014). Visnagin protects against doxorubicin-induced cardiomyopathy through modulation of mitochondrial malate dehydrogenase. Sci. Transl. Med..

[79] Xi L. (2016). Visnagin-a new protectant against doxorubicin cardiotoxicity? Inhibition of mitochondrial malate dehydrogenase 2 (MDH2) and beyond. Ann. Transl. Med..

[80] Huan M., Cui H., Teng Z., Zhang B., Wang J., Liu X., Xia H., Zhou S., Mei Q. (2012). In vivo anti-tumor activity of a new doxorubicin conjugate via alpha-linolenic acid. Biosci. Biotechnol. Biochem..

[81] Yu X., Cui L., Zhang Z., Zhao Q., Li S. (2013). alpha-Linolenic acid attenuates doxorubicin-induced cardiotoxicity in rats through suppression of oxidative stress and apoptosis. Acta Biochim. Biophys. Sin..

[82] Yang L., Luo C., Chen C., Wang X., Shi W., Liu J. (2016). All-trans retinoic acid protects against doxorubicin-induced cardiotoxicity by activating the ERK2 signalling pathway. Br. J. Pharmacol..

[83] Khafaga A.F., El-Sayed Y.S. (2018). All-trans-retinoic acid ameliorates doxorubicin-induced cardiotoxicity: In vivo potential involvement of oxidative stress, inflammation, and apoptosis via caspase-3 and p53 down-expression. Naunyn-Schmiedeberg’s Arch. Pharmacol..

[84] Zhao X.X., Guan L., Lee K., Cheng X.W., Kim W. (2016). GW27-e1068 The Improved Cell-Autonomy Role of Bay60 2770 in Doxorubicin-Cardiotoxicity Mediated by Up-Regulated Mitochondrial Ferritin and Balancing p-P53ser15: An omen of a New Hypothesis of Innovative Antitumor Approach to Cancer Therapy with Doxorubicin. J. Am. Coll. Cardiol..

[85] Zhao X., Guan L. (2017). GW28-e0180 Bay60-2770 attenuates doxorubicin cardiotoxicity by prevention of mitochondria membrane potential loss. J. Am. Coll. Cardiol..

[86] Xu Z., Lin S., Wu W., Tan H., Wang Z., Cheng C., Lu L., Zhang X. (2008). Ghrelin prevents doxorubicin-induced cardiotoxicity through TNF-alpha/NF-kappaB pathways and mitochondrial protective mechanisms. Toxicology.

[87] Wang X., Wang X.L., Chen H.L., Wu D., Chen J.X., Wang X.X., Li R.L., He J.H., Mo L., Cen X. (2014). Ghrelin inhibits doxorubicin cardiotoxicity by inhibiting excessive autophagy through AMPK and p38-MAPK. Biochem. Pharmacol..

[88] Nonaka M., Kurebayashi N., Murayama T., Sugihara M., Terawaki K., Shiraishi S., Miyano K., Hosoda H., Kishida S., Kangawa K. (2017). Therapeutic potential of ghrelin and des-acyl ghrelin against chemotherapy-induced cardiotoxicity. Endocr. J..

[89] Govender Y. (2017). Mitochondrial Catastrophe during Doxorubicin-Induced Cardiotoxicity: An Evaluation of the Protective Role of Melatonin. Ph.D. Thesis.

[90] Guven C., Taskin E., Akcakaya H. (2016). Melatonin Prevents Mitochondrial Damage Induced by Doxorubicin in Mouse Fibroblasts Through Ampk-Ppar Gamma-Dependent Mechanisms. Med. Sci. Monit. Int. Med. J. Exp. Clin. Res..

[91] Wang L., Zhang X., Chan J.Y., Shan L., Cui G., Cui Q., Wang Y., Li J., Chen H., Zhang Q. (2016). A Novel Danshensu Derivative Prevents Cardiac Dysfunction and Improves the Chemotherapeutic Efficacy of Doxorubicin in Breast Cancer Cells. J. Cell. Biochem..

[92] Gharanei M., Hussain A., Janneh O., Maddock H. (2013). Attenuation of doxorubicin-induced cardiotoxicity by mdivi-1: A mitochondrial division/mitophagy inhibitor. PLoS ONE.

[93] Givvimani S., Munjal C., Tyagi N., Sen U., Metreveli N., Tyagi S.C. (2012). Mitochondrial division/mitophagy inhibitor (Mdivi) ameliorates pressure overload induced heart failure. PLoS ONE.

[94] Zhou G.Y., Zhao B.L., Hou J.W., Ma G.E., Xin W.J. (1999). Protective effects of sodium tanshinone IIA sulphonate against adriamycin-induced lipid peroxidation in mice hearts in vivo and in vitro. Pharmacol. Res..

[95] Zhou G., Jiang W., Zhao Y., Ma G.E., Li S., Xin W., Zhao B. (2002). Interaction between sodium tanshinone IIA sulfonate and the adriamycin semiquinone free radical: A possible mechanism for antagonizing adriamycin-induced cardiotoxity. Res. Chem. Intermediat..

[96] Sishi B.J., Loos B., van Rooyen J., Engelbrecht A.M. (2013). Autophagy upregulation promotes survival and attenuates doxorubicin-induced cardiotoxicity. Biochem. Pharmacol..

[97] Garlid K.D., Paucek P., Yarov-Yarovoy V., Murray H.N., Darbenzio R.B., D’Alonzo A.J., Lodge N.J., Smith M.A., Grover G.J. (1997). Cardioprotective effect of diazoxide and its interaction with mitochondrial ATP-sensitive K+ channels. Possible mechanism of cardioprotection. Circ. Res..

[98] Hole L.D., Larsen T.H., Fossan K.O., Lime F., Schjott J. (2014). Diazoxide protects against doxorubicin-induced cardiotoxicity in the rat. BMC Pharmacol. Toxicol..

[99] Pecoraro M., Ciccarelli M., Fiordelisi A., Iaccarino G., Pinto A., Popolo A. (2018). Diazoxide Improves Mitochondrial Connexin 43 Expression in a Mouse Model of Doxorubicin-Induced Cardiotoxicity. Int. J. Mol. Sci..

[100] Lebrecht D., Geist A., Ketelsen U.P., Haberstroh J., Setzer B., Walker U.A. (2007). Dexrazoxane prevents doxorubicin-induced long-term cardiotoxicity and protects myocardial mitochondria from genetic and functional lesions in rats. Br. J. Pharmacol..

[101] QuanJun Y., GenJin Y., LiLi W., YongLong H., Yan H., Jie L., JinLu H., Jin L., Run G., Cheng G. (2017). Protective Effects of Dexrazoxane against Doxorubicin-Induced Cardiotoxicity: A Metabolomic Study. PLoS ONE.

[102] Asensiolopez M.C., Sanchezmas J., Pascualfigal D.A., Abenza S., Perezmartinez M.T., Pastorperez F., Garridobravo I., Valdeschavarri M., Lax A.M. (2013). Doxorubicin induced cardiotoxicity is attenuated by metformin through improvements in mitochondrial stabilization. Eur. Heart J..

[103] El-Ashmawy N.E., Khedr N.F., El-Bahrawy H.A., Abo Mansour H.E. (2017). Metformin augments doxorubicin cytotoxicity in mammary carcinoma through activation of adenosine monophosphate protein kinase pathway. Tumour Biol. J. Int. Soc. Oncodev. Biol. Med..

[104] Li Y., Wang M., Zhi P., You J., Gao J.Q. (2018). Metformin synergistically suppress tumor growth with doxorubicin and reverse drug resistance by inhibiting the expression and function of P-glycoprotein in MCF7/ADR cells and xenograft models. Oncotarget.

[105] Abdel-Raheem I.T., Taye A., Abouzied M.M. (2013). Cardioprotective effects of nicorandil, a mitochondrial potassium channel opener against doxorubicin-induced cardiotoxicity in rats. Basic Clin. Pharmacol. Toxicol..

[106] Ahmed L.A., El-Maraghy S.A. (2013). Nicorandil ameliorates mitochondrial dysfunction in doxorubicin-induced heart failure in rats: Possible mechanism of cardioprotection. Biochem. Pharmacol..

[107] Asensio-Lopez M.C., Soler F., Pascual-Figal D., Fernandez-Belda F., Lax A. (2017). Doxorubicin-induced oxidative stress: The protective effect of nicorandil on HL-1 cardiomyocytes. PLoS ONE.

[108] Das A., Xi L., Kukreja R.C. (2005). Phosphodiesterase-5 inhibitor sildenafil preconditions adult cardiac myocytes against necrosis and apoptosis. Essential role of nitric oxide signaling. J. Biol. Chem..

[109] Koka S., Kukreja R.C. (2010). Attenuation of Doxorubicin-induced Cardiotoxicity by Tadalafil: A Long Acting Phosphodiesterase-5 Inhibitor. Mol. Cell. Pharmacol..

[110] Greish K., Fateel M., Abdelghany S., Rachel N., Alimoradi H., Bakhiet M., Alsaie A. (2017). Sildenafil citrate improves the delivery and anticancer activity of doxorubicin formulations in a mouse model of breast cancer. J. Drug Target..

[111] Yaidikar L., Thakur S. (2015). Arjunolic acid, a pentacyclic triterpenoidal saponin of Terminalia arjuna bark protects neurons from oxidative stress associated damage in focal cerebral ischemia and reperfusion. Pharmacol. Rep..

[112] Ghosh J., Das J., Manna P., Sil P.C. (2010). Arjunolic acid, a triterpenoid saponin, prevents acetaminophen (APAP)-induced liver and hepatocyte injury via the inhibition of APAP bioactivation and JNK-mediated mitochondrial protection. Free Radic. Biol. Med..

[113] Masoko P., Mdee L.K., Mampuru L.J., Eloff J.N. (2008). Biological activity of two related triterpenes isolated from *Combretum nelsonii* (Combretaceae) leaves. Nat. Prod. Res..

[114] Lau C.W., Yao X.Q., Chen Z.Y., Ko W.H., Huang Y. (2001). Cardiovascular actions of berberine. Cardiovasc. Ther..

[115] El-Sayed E.M., El-Azeem A.S.A., Afify A.A., Shabana M.H., Ahmed H.H. (2011). Cardioprotective effects of Curcuma longa L. extracts against doxorubicin-induced cardiotoxicity in rats. J. Med. Plant Res..

[116] Zhou L., Zuo Z., Chow M.S. (2005). Danshen: An overview of its chemistry, pharmacology, pharmacokinetics, and clinical use. J. Clin. Pharmacol..

[117] Nabavi S.F., Braidy N., Habtemariam S., Orhan I.E., Daglia M., Manayi A., Gortzi O., Nabavi S.M. (2015). Neuroprotective effects of chrysin: From chemistry to medicine. Neurochem. Int..

[118] Walle T., Otake Y., Brubaker J.A., Walle U.K., Halushka P.V. (2001). Disposition and metabolism of the flavonoid chrysin in normal volunteers. Br. J. Clin. Pharmacol..

[119] Yu B., Fang T.H., Lu G.H., Xu H.Q., Lu J.F. (2011). Beneficial effect of Cyclovirobuxine D on heart failure rats following myocardial infarction. Fitoterapia.

[120] Mukhopadhyay P., Hao E., Cao Z., Holovac E., Erdelyi K., Pacher P. (2013). PSS163–Cannabidiol Attenuates Cardiac Dysfunction, Oxidative Stress, and Cell Death in Doxorubicin Induced Cardiomyopathy. Free Radic. Biol. Med..

[121] Fouad A.A., Albuali W.H., Al-Mulhim A.S., Jresat I. (2013). Cardioprotective effect of cannabidiol in rats exposed to doxorubicin toxicity. Environ. Toxicol. Pharmacol..

[122] Park C., Jin C.Y., Kim G.Y., Choi I.W., Kwon T.K., Choi B.T., Lee S.J., Lee W.H., Choi Y.H. (2008). Induction of apoptosis by esculetin in human leukemia U937 cells through activation of JNK and ERK. Toxicol. Appl. Pharmacol..

[123] Subramaniam S.R., Ellis E.M. (2013). Neuroprotective effects of umbelliferone and esculetin in a mouse model of Parkinson’s disease. J. Neurosci. Res..

[124] Bulotta S., Celano M., Lepore S.M., Montalcini T., Pujia A., Russo D. (2014). Beneficial effects of the olive oil phenolic components oleuropein and hydroxytyrosol: Focus on protection against cardiovascular and metabolic diseases. J. Transl. Med..

[125] Granados-Principal S., Quiles J.L., Ramirez-Tortosa C.L., Sanchez-Rovira P., Ramirez-Tortosa M.C. (2010). Hydroxytyrosol: From laboratory investigations to future clinical trials. Nutr. Rev..

[126] Devi K.P., Malar D.S., Nabavi S.F., Sureda A., Xiao J., Nabavi S.M., Daglia M. (2015). Kaempferol and inflammation: From chemistry to medicine. Pharmacol. Res..

[127] Vellosa J.C.R., Regasini L.O., Khalil N.M., Bolzani V.D.S., Khalil O.A.K., Manente F.A., Netto H.P., Oliveira O.M. (2011). Antioxidant and cytotoxic studies for kaempferol, quercetin and isoquercitrin. Eclética Química.

[128] Lee J., Kim J.H. (2016). Kaempferol Inhibits Pancreatic Cancer Cell Growth and Migration through the Blockade of EGFR-Related Pathway In Vitro. PLoS ONE.

[129] Wang S.Q., Han X.Z., Li X., Ren D.M., Wang X.N., Lou H.X. (2010). Flavonoids from Dracocephalum tanguticum and their cardioprotective effects against doxorubicin-induced toxicity in H9c2 cells. Bioorg. Med. Chem. Lett..

[130] Shimosaki S., Tsurunaga Y., Itamura H., Nakamura M. (2011). Anti-allergic effect of the flavonoid myricitrin from Myrica rubra leaf extracts in vitro and in vivo. Nat. Prod. Res..

[131] Fernandez S.P., Nguyen M., Yow T.T., Chu C., Johnston G.A., Hanrahan J.R., Chebib M. (2009). The flavonoid glycosides, myricitrin, gossypin and naringin exert anxiolytic action in mice. Neurochem. Res..

[132] Chandra G., Jagetia M.U. (2014). The grape fruit flavonone naringin protects mice against doxorubicin-induced cardiotoxicity. J. Mol. Biochem..

[133] Jagetia G.C., Lalrinengi C. (2017). Treatment of mice with naringin alleviates the doxorubicin-induced oxidative stress in the liver of swiss albino mice. MOJ Anat. Physiol..

[134] Xiao T.T., Wang Y.Y., Zhang Y., Bai C.H., Shen X.C. (2014). Similar to spironolactone, oxymatrine is protective in aldosterone-induced cardiomyocyte injury via inhibition of calpain and apoptosis-inducing factor signaling. PLoS ONE.

[135] Qian J., Jiang F., Wang B., Yu Y., Zhang X., Yin Z., Liu C. (2010). Ophiopogonin D prevents H_2_O_2_-induced injury in primary human umbilical vein endothelial cells. J. Ethnopharmacol..

[136] Serban M.C., Sahebkar A., Zanchetti A., Mikhailidis D.P., Howard G., Antal D., Andrica F., Ahmed A., Aronow W.S., Muntner P. (2016). Effects of Quercetin on Blood Pressure: A Systematic Review and Meta-Analysis of Randomized Controlled Trials. J. Am. Heart Assoc..

[137] Helli B., Mowla K., Mohammadshahi M., Jalali M.T. (2016). Effect of Sesamin Supplementation on Cardiovascular Risk Factors in Women with Rheumatoid Arthritis. J. Am. Coll. Nutr..

[138] Ma J.Q., Ding J., Zhang L., Liu C.M. (2014). Hepatoprotective properties of sesamin against CCl_4_ induced oxidative stress-mediated apoptosis in mice via JNK pathway. Food Chem. Toxicol. Int. J. Publ. Br. Ind. Biol. Res. Assoc..

[139] Liang Y.T., Chen J., Jiao R., Peng C., Zuo Y., Lei L., Liu Y., Wang X., Ma K.Y., Huang Y. (2015). Cholesterol-lowering activity of sesamin is associated with down-regulation on genes of sterol transporters involved in cholesterol absorption. J. Agric. Food Chem..

[140] Buchter C., Zhao L., Havermann S., Honnen S., Fritz G., Proksch P., Watjen W. (2015). TSG (2,3,5,4′-Tetrahydroxystilbene-2-*O*-beta-d-glucoside) from the Chinese Herb Polygonum multiflorum Increases Life Span and Stress Resistance of Caenorhabditis elegans. Oxid. Med. Cell. Longev..

[141] Wang X., Zhao L., Han T., Chen S., Wang J. (2008). Protective effects of 2,3,5,4′-tetrahydroxystilbene-2-*O*-beta-d-glucoside, an active component of Polygonum multiflorum Thunb, on experimental colitis in mice. Eur. J. Pharmacol..

[142] Anrep G.V., Barsoum G.S., Kenawy M.R., Misrahy G. (1946). Ammi Visnaga in the Treatment of the Anginal Syndrome. Br. Heart J..

[143] Mozaffarian D., Wu J.H. (2011). Omega-3 fatty acids and cardiovascular disease: Effects on risk factors, molecular pathways, and clinical events. J. Am. Coll. Cardiol..

[144] Kukoba T.V., Shysh A.M., Moibenko O.O., Kotsiuruba A.V., Kharchenko O.V. (2006). The effects of alpha-linolenic acid on the functioning of the isolated heart during acute myocardial ischemia/reperfusion. Fiziolohichnyi Zhurnal.

[145] Sun R., Liu Y., Li S.Y., Shen S., Du X.J., Xu C.F., Cao Z.T., Bao Y., Zhu Y.H., Li Y.P. (2015). Co-delivery of all-trans-retinoic acid and doxorubicin for cancer therapy with synergistic inhibition of cancer stem cells. Biomaterials.

[146] Zhang T., Xiong H., Dahmani F.Z., Sun L., Li Y., Yao L., Zhou J., Yao J. (2015). Combination chemotherapy of doxorubicin, all-trans retinoic acid and low molecular weight heparin based on self-assembled multi-functional polymeric nanoparticles. Nanotechnology.

[147] Alexandre E.C., Leiria L.O., Silva F.H., Mendes-Silverio C.B., Calmasini F.B., Davel A.P., Monica F.Z., De Nucci G., Antunes E. (2014). Soluble guanylyl cyclase (sGC) degradation and impairment of nitric oxide-mediated responses in urethra from obese mice: Reversal by the sGC activator BAY 60-2770. J. Pharmacol. Exp. Ther..

[148] Ledderose C., Kreth S., Beiras-Fernandez A. (2011). Ghrelin, a novel peptide hormone in the regulation of energy balance and cardiovascular function. Recent Pat. Endocr. Metab. Immune Drug Discov..

[149] Sato T., Nakamura Y., Shiimura Y., Ohgusu H., Kangawa K., Kojima M. (2012). Structure, regulation and function of ghrelin. J. Biochem..

[150] Zhang Y., Li L., Xiang C., Ma Z., Ma T., Zhu S. (2013). Protective effect of melatonin against Adriamycin-induced cardiotoxicity. Exp. Ther. Med..

[151] Cassidy-Stone A., Chipuk J.E., Ingerman E., Song C., Yoo C., Kuwana T., Kurth M.J., Shaw J.T., Hinshaw J.E., Green D.R. (2008). Chemical inhibition of the mitochondrial division dynamin reveals its role in Bax/Bak-dependent mitochondrial outer membrane permeabilization. Dev. Cell.

[152] Cheng J., Chen T., Li P., Wen J., Pang N., Zhang L., Wang L. (2018). Sodium tanshinone IIA sulfonate prevents lipopolysaccharide-induced inflammation via suppressing nuclear factor-kappaB signaling pathway in human umbilical vein endothelial cells. Can. J. Physiol. Pharmacol..

[153] Zhang M.Q., Zheng Y.L., Chen H., Tu J.F., Shen Y., Guo J.P., Yang X.H., Yuan S.R., Chen L.Z., Chai J.J. (2013). Sodium tanshinone IIA sulfonate protects rat myocardium against ischemia-reperfusion injury via activation of PI3K/Akt/FOXO3A/Bim pathway. Acta Pharmacol. Sin..

[154] Jiang B., Zhang L., Wang Y., Li M., Wu W., Guan S., Liu X., Yang M., Wang J., Guo D.A. (2009). Tanshinone IIA sodium sulfonate protects against cardiotoxicity induced by doxorubicin in vitro and in vivo. Food Chem. Toxicol. Int. J. Publ. Br. Ind. Biol. Res. Assoc..

[155] Ghosh R., Pattison J.S. (2018). Macroautophagy and Chaperone-Mediated Autophagy in Heart Failure: The Known and the Unknown. Oxid. Med. Cell. Longev..

[156] Coetzee W.A. (2013). Multiplicity of effectors of the cardioprotective agent, diazoxide. Pharmacol. Ther..

[157] Liesse K., Harris J., Chan M., Schmidt M.L., Chiu B. (2018). Dexrazoxane Significantly Reduces Anthracycline-induced Cardiotoxicity in Pediatric Solid Tumor Patients: A Systematic Review. J. Pediatr. Hematol./Oncol..

[158] Emeka P.M., Al-Ahmed A. (2017). Effect of metformin on ECG, HR and BP of rats administered with cardiotoxic agent doxorubicin. Int. J. Basic Chin. Pharmacol..

[159] Afzal M.Z., Reiter M., Gastonguay C., McGivern J.V., Guan X., Ge Z.D., Mack D.L., Childers M.K., Ebert A.D., Strande J.L. (2016). Nicorandil, a Nitric Oxide Donor and ATP-Sensitive Potassium Channel Opener, Protects Against Dystrophin-Deficient Cardiomyopathy. J. Cardiovasc. Pharmacol. Ther..

[160] Varga Z.V., Ferdinandy P., Liaudet L., Pacher P. (2015). Drug-induced mitochondrial dysfunction and cardiotoxicity. Am. J. Physiol. Heart Circ. Physiol..

